# Sleep health epidemiology and associations with menstrual health, mental health, and educational performance among in-school female adolescents in Uganda: A longitudinal study

**DOI:** 10.1016/j.sleh.2024.12.007

**Published:** 2025-01-30

**Authors:** Beatrice Nanyonga, Katherine A. Thomas, Titus Ssesanga, Apophia Kaihangwe, Kate A. Nelson, Denis Ssenyondwa, Noeline Nassimbwa, Jonathan Reuben Enomut, Aggrey Tumuhimbise, Prossy Namirembe, Ratifah Batuusa, Nambusi Kyegombe, Fiona C. Baker, Helen A. Weiss

**Affiliations:** a MRC/UVRI and LSHTM Uganda Research Unit, Entebbe, Uganda; b MRC International Statistics and Epidemiology Group, London School of Hygiene & Tropical Medicine, London, United Kingdom; c Center for Health Sciences, SRI International, Menlo Park, California, USA; d School of Physiology, University of the Witwatersrand, Johannesburg, South Africa

**Keywords:** Sleep health, Menstrual health, Mental health, Education, Adolescents, Uganda

## Abstract

**Objectives:**

Few studies have assessed sleep among African adolescents. We aim to understand factors associated with subjective sleep quality among female Ugandan adolescents and the association of poor sleep quality with subsequent menstrual- and mental health, and educational performance.

**Methods:**

We analyzed data from a cluster-randomized controlled trial that evaluated a menstrual health intervention in 60 Ugandan secondary schools. Data were collected through cross-sectional surveys at baseline (March-June 2022) and endline (July-August 2023), and prospective daily diaries (April-August 2023). We used logistic regression to analyze associations with poor sleep at baseline, and linear regression to analyze associations of poor sleep with subsequent menstrual and mental health, and education performance, adjusting for clustering.

**Results:**

Of 3841 female participants (mean age = 15.6 years), 580 (15.1%) reported poor sleep quality and 829 (21.6%) reported feeling tired at baseline. Poor sleep was associated with socio-economic factors including smaller household size, lower socioeconomic status, and fewer meals consumed the previous day. There was strong evidence that poor sleep at baseline was associated with multiple dimensions of poor menstrual health including menstrual pain (adjusted odds ratio = 1.74, 95%CI 1.29-2.33), more unmet menstrual practice needs (adjusted odds ratio = 2.68, 95%CI 1.99-3.60), and with mental health problems (adjusted odds ratio=2.40, 95%CI 1.80-3.19). Results were similar for baseline tiredness. Prospectively reported poor sleep quality was associated with subsequent poor menstrual and mental health, and subsequent poor educational performance.

**Conclusions:**

Poor sleep is prevalent among in-school female Ugandan adolescents and is associated with subsequent poorer menstrual health, mental health, and educational performance. Improving sleep in this population could benefit menstrual health, mental health and education outcomes.

## Introduction

Sleep problems are an increasing public health concern,^1^ and poor sleep during adolescence can adversely affect physical and mental health, functioning, educational performance, and risk-taking.^2–5^ Poor sleep is common in adolescents due to a biological shift to evening chronotype and physiological changes in sleep homeostasis,^6^ socio-behavioral pressures, early school start times,^3^ and increased risk for insomnia disorder.^7^ The Global School-based Health Survey (GSHS) survey among 483,098 13-17 year-olds in 98 countries found an average prevalence of sleep disturbances of 9.6%.^1^

Improving the health and education of adolescents in African countries is critically important for the Sustainable Development Goals, as 40% of people aged under 18 years will be living in Africa by 2050.^8^ Poor sleep is associated with increasing public health issues in many African settings, including mental health problems and noncommunicable diseases.^9^ Poor sleep among adolescents in the region is likely to increase with smartphone use^10^ and climate-related factors through increased stress and trauma, displacement, food insecurity, and higher temperatures.^11^ The GSHS survey shows a high prevalence of sleep disturbance in 14 African countries with data (average 15.2%)^1^ but there are few epidemiological studies of sleep among adolescents and young people in African countries. A systematic review of sleep among university students identified 35 studies from 11 African countries with a pooled prevalence of poor sleep quality of 63.3% (95%CI 56.9%-65.7%) mainly using the Pittsburgh Sleep Quality Index (PSQI).^12^

Globally among adolescents, females are more likely to have insomnia disorder (the most severe clinical manifestation of sleep dissatisfaction) than males.^7^ Females also commonly face menstrual health challenges,^13^ and systematic reviews have shown that poor sleep is associated with premenstrual syndrome, menstrual pain (dysmenorrhea), and heavy bleeding.^14,15^ However, almost all studies in these systematic reviews were cross-sectional or case-control and only 2 were from sub-Saharan Africa.^15^

Sleep and mental health have a bidirectional relationship, with biopsychosocial mechanisms interacting to increase vulnerability to sleep problems and internalizing disorders.^16–19^ A recent meta-analysis of longitudinal studies on sleep, mental health, and well-being in adolescents highlighted the need for longitudinal epidemiological studies to disentangle the bidirectional effect and found few studies from low- and middle-income countries (LMICs), and no studies from African countries.^18^ Poor sleep also affects memory and cognition, and the meta-analysis of longitudinal studies on sleep and school experience identified 25 studies (none from African countries) with only seven studies assessing the association of poor sleep with examination performance.^18^ Of these, five found that sleep disturbances or short sleep duration were associated with poorer examination performance. Poor sleep quality was also bi-directionally associated with higher levels of school burnout, lower levels of school connectedness, and higher levels of school bullying.^18^

Our aim is to address the knowledge gap on the epidemiology of sleep health among female adolescents living in Uganda including longitudinal associations of poor sleep with subsequent menstrual health, mental health, and educational performance. The objectives are to investigate (i) cross-sectional associations between subjective sleep quality and tiredness at baseline with socio-demographic, menstrual- and mental health-related factors, (ii) longitudinal associations between subjective sleep quality and subsequent menstrual health, mental health and school examination performance as outcomes at 1 year of follow-up, and (iii) longitudinal associations between daily reported sleep and daily menstrual characteristics, during a 16-week period prior to endline survey.

## Participants and methods

### Study design and setting

This study is a secondary analysis of data from the MENISCUS trial (registration number ISRCTN45461276), a cluster-randomized trial that evaluated the impact of a multicomponent menstrual health intervention in Ugandan secondary schools on girls’ education, health, and well-being.^20^ Uganda’s education system comprises 7 years of primary education, 4 years of lower secondary (Secondary 1-Secondary 4), and 2 years of upper secondary (Secondary 5 and 6). English is the official language for instruction and assessments and students in lower secondary take science subjects including biology, chemistry, physics, and mathematics.^21^ The trial was conducted in 60 schools in two districts in central Uganda (44 schools in Wakiso District; and 16 in Kalungu District). Wakiso district includes urban and rural areas and partly encircles Kampala (Uganda’s capital) while Kalungu district is largely rural.

### Study population

Schools were eligible for inclusion if they were mixed day/boarding or day secondary schools; had an estimated enrollment of around 50-150 students in Secondary 1 in Wakiso and 40-125 students in Kalungu; and at least with minimal water, sanitation, and hygiene facilities.^22^ All female participants in Secondary 1 in 2021 were eligible for the study. The baseline survey was conducted in 2022, by which time the participants were in Secondary 2. Parental consent was sought for eligible female students under ≤18, with electronic written informed assent from students. Students aged ≥18 years provided written informed consent. Ethics approval for the trial was granted by the Uganda Virus Research Institute Research Ethics Committee, the Uganda National Council of Science and Technology, and the London School of Hygiene and Tropical Medicine Interventional Research Ethics Committee.

### Data collection

Participants self-completed a baseline survey using Open Data Kit (ODK) software between March-July 2022, and an endline survey between June-August 2023. Surveys were conducted at school, using tablets and headphones provided by the research team. Questions were administered in English (the language of instruction in Ugandan schools). Selected keywords were translated into Luganda as informed by cognitive testing. The survey included questions on socio-demographic factors, knowledge of puberty and menstruation, menstrual experiences, mental health, and sleep quality.^20^ We randomly selected 25 post-menarchal participants at each school to participate in a diary substudy in which they were asked to prospectively record daily answers to closed-ended questions on their menstrual flow, menstrual pain, use of painkillers, sleep, and school attendance in a paper diary, for about 4 months from April to August 2023.

### Assessment of subjective sleep quality

We assessed subjective sleep quality through baseline and endline surveys with the questions; “How well did you sleep last night” (referred to as “sleep quality”), and “How tired are you feeling today” (“tiredness”). Response options were on a 5-point Likert scale from “no problems sleeping” to “I couldn’t sleep at all,” and from “I don’t feel tired today” to “I feel very tired today.” We defined participants as having poor sleep quality if they reported having “some” or “many” problems, or that they “couldn’t sleep at all,” and defined tiredness as reporting feeling “a bit” “quite” or “very” tired today. Sensitivity analyses were conducted with a stricter definition of poor sleep quality defined as “many” problems or “couldn’t sleep at all” and tiredness as “quite” or “very” tired. The diary substudy participants responded each day to the question “How was your sleep last night?” with response options “poor,” “fair,” or “good.”

### Statistical methods

#### Research question 1: What are the associations between subjective sleep quality and tiredness at baseline with socio-demographic, menstrual- and mental health-related factors?

**Fig. 1:**
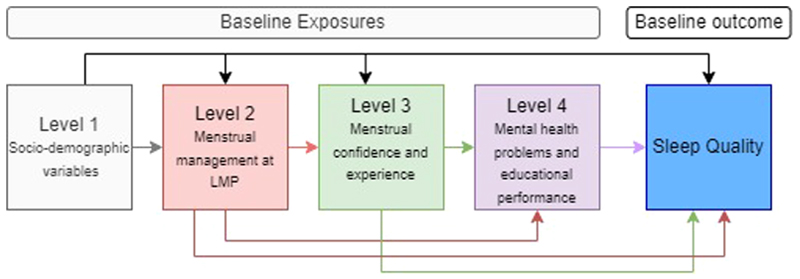
Conceptual framework for analyses of factors associated with sleep quality at baseline

In these cross-sectional analyses, outcomes were baseline sleep quality and tiredness respectively, and the exposures were baseline socio-demographic (level 1), menstrual health (levels 2 and 3), and mental health symptoms (level 4) (Fig. 1). Menstrual management variables at last menstrual period (LMP) for participants who reported menstruating in the past 6 months were: pain management at LMP (defined as “no pain,” “pain with effective pain management,” and “pain with no effective pain management”), pain relief, and type of menstrual product used (disposable only, reusable only or both) (level 2). Effective pain management was defined as using at least one effective method (painkillers, exercising, drinking lots of clean water, stretching, using a warm water bottle, and eating foods containing lots of water), and none of the ineffective methods (doing nothing, taking antibiotics, eating spicy foods, and drinking soda). Level 3 variables assessed menstrual experience at the LMP: experience of menstrual teasing, the Menstrual Practice Needs Scale (MPNS) score which measures participants’ perceptions of their menstrual needs,^23^ and the Self-efficacy in addressing Menstrual Needs Scale (SAMNS) which measures a participant’s confidence in her capabilities to address her menstrual needs.^24^ We categorized the mean MPNS-36 and SAMNS-26 scores into tertiles of low, medium, and high, with lower scores corresponding to more unmet menstrual needs and poorer confidence in managing menstruation. Level 4 variables assessed mental health by reported anxiety about the next period and the Strengths and Difficulties Questionnaire (SDQ) Total Difficulties score, which has been used widely among adolescents in African countries to measure behavioral and emotional difficulties.^25^ School performance was assessed by examinations set by the Uganda National Examination Board (UNEB) (mathematics and biology at baseline, mathematics, biology, and English at endline). English was excluded from the baseline examination for logistical reasons as it is more time-intensive to administer and mark, and the primary purpose of the baseline assessment was to inform the restricted randomization for the trial.

We used mixed-effects logistic regression models to estimate adjusted odds ratios (aOR) and 95% confidence intervals (CI) for the associations of poor sleep quality and tiredness respectively with exposures as shown in Fig. 1. We fitted multivariable models adjusting each exposure variable for other variables on that level and more distal levels.

#### Research question 2: What are the longitudinal associations between menstrual health (MPNS and SAMNS scores), mental health (SDQ score) and examination performance (UNEB score) as outcomes at endline, with prior subjective sleep quality

**Fig. 2:**
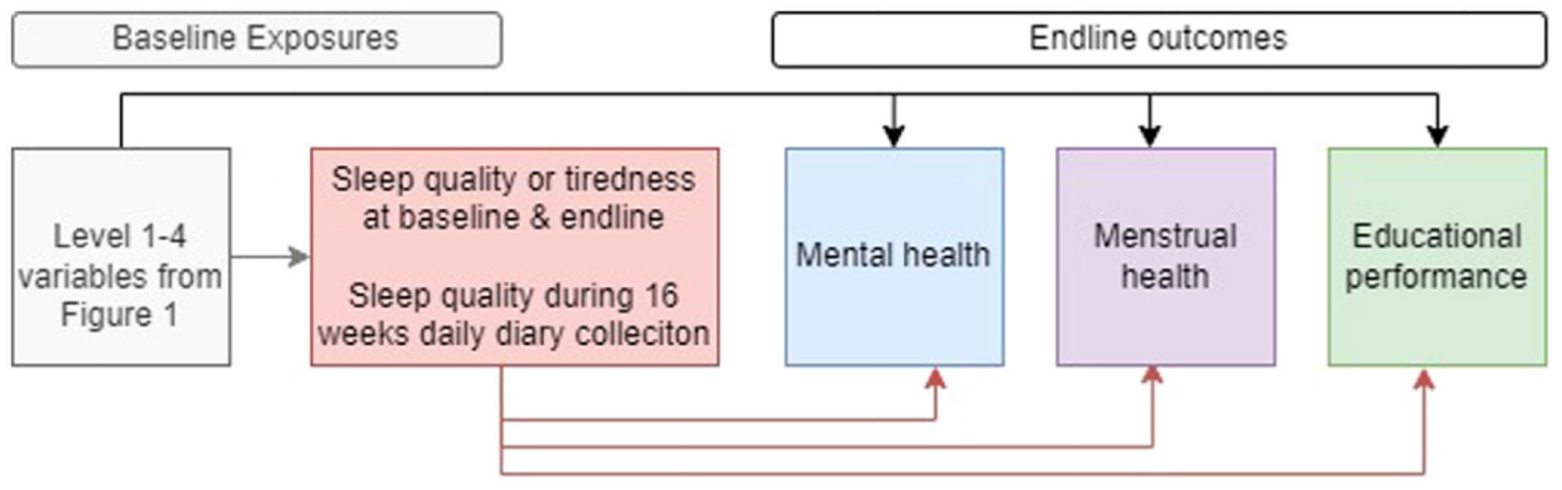
Conceptual framework for analyses of sleep associated with subsequent menstrual health, mental health, and educational performance

We estimated associations between each of the four outcomes (MPNS score, SAMNS score, SDQ score, UNEB score) and persistent sleep problems by categorizing sleep quality and tiredness based on baseline and endline survey responses (e.g., no sleep problems at either time point, sleep problems at baseline only, sleep problems at endline only, sleep problems at both time points, (Fig. 2). We used mixed-effects linear regression to estimate adjusted mean differences (aMD) and adjusted standardized mean differences (aSMD) for each outcome, adjusted for baseline potential confounders (all variables in Fig. 1) and within-school clustering. We further estimated associations between each outcome with the proportion of days on which poor sleep quality was reported in the diaries prior to the endline survey (none, 1%-9%; 10%-19%, ≥20%), using per-participant mixed-effects linear regression to estimate the aMDs and aSMDs, adjusting for socio-economic factors and the baseline value of the outcome variable.

#### Research question 3: What are the associations between daily reported sleep and daily menstrual characteristics?

For this research question, the outcome was daily recorded sleep quality from the diaries over 16 weeks (daily entry of poor vs. fair/good quality), and exposures were daily recorded menstrual flow, period pain, and school attendance. We estimated aORs using per-day mixed-effects logistic regression adjusted for baseline confounders and within-school and within-individual correlation.

## Results

Of the 4281 female students eligible for the baseline survey, 399 (9.3%) were not enrolled due to lack of parental consent (n = 238, 5.6%), student assent (n = 12, 0.28%), or absence at baseline survey (n = 149, 3.5%). The mean age of the remaining 3841 participants was 15.6 years (SD 0.95), of whom 55.3% were day students (Table 1). Diaries were distributed to 1477 substudy participants in April 2023, of whom 1332 (90.2%) returned data (median 115 days with sleep data completed [IQR 109-120]). Participants reported sleep data for a median of 66 days (IQR 62-79) prior to completing the endline survey. The scales for menstrual health had strong internal consistency (MPNS: Cronbach’s alpha = 0.92; SAMNS: Cronbach’s alpha = 0.90), and the SDQ had good internal consistency (Cronbach’s alpha = 0.75).

### Associations between subjective sleep quality and tiredness at baseline with socio-demographic, menstrual- and mental health-related factors

Of the 3841 participants, 580 (15.1%) reported poor sleep quality the previous night, and 829 (21.6%) reported tiredness that day. Overall, 1159 (30.2%) participants reported either poor sleep quality or tiredness, and 579 (15.1%) reported tiredness but no problems sleeping. Socio-demographic factors associated with poor sleep quality the previous night were being in a small household (aOR = 1.29, 95%CI 1.03-1.60 for ≤5 vs. ≥8 household members), low socio-economic status (aOR = 1.58, 95%CI 1.17-2.12 for lowest vs. highest SES category) and reporting few meals eaten the previous day (aOR = 2.09, 95% CI 1.61-2.70 for ≤1 vs. ≥3 meals) (Table 1). After adjusting for socio-demographic factors, there was strong evidence that poor sleep quality was associated with menstrual health including pain at LMP (aOR = 1.74, 95%CI 1.29-2.33 for pain with no effective management vs. no pain), using reusable products only compared to using disposable products only (aOR = 2.23, 95%CI 1.73-2.87), reporting menstrual teasing (aOR = 1.57, 95%CI 1.21-2.04), more unmet menstrual practice needs (MPNS score: aOR = 2.68, 95%CI 1.99-3.60 for lowest vs. highest tertile) and poorer menstrual self-efficacy (SAMNS score: aOR = 1.40, 95%CI 1.07-1.83 for lowest vs. highest tertile) (Table 1). Finally, there was strong evidence that participants with poor sleep were more likely to report being anxious about their next period (aOR = 1.62, 95%CI 1.28-2.06) and have more mental health problems (aOR = 2.40, 95%CI 1.80-3.19 for highest vs. lowest category of the SDQ).

Tiredness was associated with additional socio-demographic factors including being in a larger school (aOR = 1.23, 95%CI 1.03-1.48), being a boarding student (aOR = 1.28, 95%CI 1.06-1.54) and being younger (aOR = 0.61, 95%CI 0.35-1.05 for age ≥18 vs. ≤15 years) (Table 1). Unlike for poor sleep quality, there was little evidence of an association that tiredness was associated with household size or socio-economic status. There was strong and consistent evidence that tiredness was associated with multiple dimensions of poor menstrual health and mental health problems (Table 1). Results of the sensitivity analyses with the stricter definition of poor sleep quality and tiredness were similar (Table A1).

### Longitudinal associations between sleep quality with endline menstrual health (MPNS and SAMNS scores), mental health (SDQ score) and examination performance (UNEB score)

Of the 2901 (75.5%) participants seen at endline, 309 (10.7%) had poor sleep at baseline only, 297 (10.2%) at endline only and 110 (3.8%) at both time points (Table 2a). There was strong evidence that persistently poor sleep quality and tiredness (i.e., at both time points) were associated with more unmet menstrual practice needs (*p* < .001), poor menstrual self-efficacy (*p* < .001) and mental health problems (*p* < .001) at endline (Tables 2a and 2b). Poor sleep quality was also associated with poorer examination performance (*p* = .02) (Table 2a), especially when using the stricter definition of poor sleep quality (*p* < .001; Table A2a).

Of the 1332 substudy participants, 1328 had sleep data prior to endline survey data. Of these, 121 (9.1%) reported having had poor sleep on ≥20% of nights during diary data collection, and 354 participants (26.7%) reported no nights with poor sleep. There was strong evidence of a trend between the increasing proportion of nights with poor sleep with more unmet menstrual health needs (aSMD = −0.39, 95%CI −0.58, −0.21 for ≥20% of nights with poor sleep vs. none; *p*-trend < .001), poorer menstrual self-efficacy (aSMD = −0.21, 95%CI −0.41, −0.01, *p*-trend = .001), more mental health problems (aSMD = 0.50, 95%CI 0.32, 0.68; *p*-trend < .001) and weaker evidence that poor sleep was associated with poorer examination performance (aSMD = −0.17, 95%CI −0.37, 0.02, *p*-trend = .05) (Table 3).

### Associations between daily reported sleep and daily menstrual characteristics

Poor sleep quality the previous night was reported on 9695/144,884 (6.9%) days among the 1332 substudy participants. Poor sleep was strongly associated with period days (16.4% vs. 5.4% on period vs. nonperiod days; aOR = 4.32, 95%CI 3.69-5.06), especially days with heavy flow (36.6%; aOR = 3.96, 95%CI 3.11-5.04) or period pain (30.5%; aOR = 5.79, 95%CI 4.71-7.11) after adjustment for each other and baseline confounders (Table 4). Participants were also more likely to report poor sleep on nights before they missed school (12.5% vs. 5.7%; aOR = 2.22, 95%CI 1.89-2.60; Table 4).

## Discussion

This study adds to the limited literature on sleep epidemiology among adolescents in LMICs. To our knowledge, this is the first paper to assess the association between menstrual health and sleep quality among adolescents in an African country, and one of few longitudinal studies on associations of sleep with mental health and educational performance in the region. Using different timepoints and data sources, we found strong and consistent associations between poor sleep and tiredness with menstrual health and mental health, and to a lesser extent with educational performance.

Two previous studies in Uganda have assessed sleep and mental health in adolescents.^26,27^ One, a cross-sectional study among 617 boarding-school students aged 12-17 years, found that 59.2% of participants reported poor sleep by the PSQI, and were less likely to have good psychological well-being (8.9% vs. 34.7%; *p* = .03).^27^ The second, a cross-sectional study among 148 rural and urban students aged 11-16 years in 2 day-only primary schools in 2013, found that 93% of participants responded “very good” when asked “During the past week, how well did you sleep?”^26^ The variability in the prevalence of sleep problems may be due to differences in age, school-related pressures, day vs. boarding status, social media use, or the use of nonvalidated tools.

The prevalence we found of 15.1% of girls reporting poor sleep quality the previous night and 21.5% reporting tiredness, is similar to GSHS survey data from African countries (15.2%).^1^ In line with the socio-ecological model for sleep,^28^ we found multiple socio-demo-graphic factors associated with poor sleep quality and/or tiredness. The increased tiredness among Muslim students may be a chance finding or may be due to the early waking of these students to pray (the endline survey was not conducted during Ramadan). Religion has been cited as an important cultural factor in sleep research, but there is little empirical evidence on how religion affects sleep patterns^29^ and further research is needed. The increased tiredness among boarding school students may be due to the dormitory environment (noise, light, temperature) and early waking.

Having fewer meals the previous day was associated with poor sleep quality and tiredness after adjusting for confounders including socio-economic status, supporting evidence from the United States that food insecurity is an independent indicator of sleep problems.^30,31^ Potential mechanisms include diet composition and eating behaviors (quantity, meal timing) affecting sleep directly through the impact on regulatory hormonal pathways^32^ but there are few studies on this in LMICs.

Further qualitative research is needed to understand the association of sleep problems with the home environment (primary caregiver, household size, and socio-economic status) in LMICs, particularly to explore how sleep quality reflects parent-child interactions, attachment and security, and the physical environment (e.g., cosleeping and use of bedrooms for nonsleeping activities). We found only one study on this in Africa, among in-school adolescents in Nigeria, in whom poor sleep was associated with living at home rather than at a hostel, possibly due to the less-regimented routine at home, or over-crowding.^33^

We saw no evidence of an association of age with reported sleep quality, but older girls (age ≥18) were less likely to feel tired than those aged ≤15 (16.3% vs. 26.1%). This contrasts with other studies which find that older adolescents tend to have poorer sleep,^34–36^ and likely reflects that our participants were all in the same school year so older age reflects factors such as repeating a school year or missing school due to inability to pay fees.

### Association of sleep with menstrual health

Associations between menstrual health and sleep health are likely bi-directional, but the exact mechanisms are unknown.^37^ In our study, poor sleep quality and tiredness were associated with pain at LMP, regardless of pain management strategy used or subjective pain relief. These findings are consistent with a systematic review of 35 studies among adult women which found that dysmenorrhea was associated with multiple dimensions of sleep,^14^ and with a review of sleep and chronic pain among adolescents, which found bi-directional associations, with stronger effects of poor sleep on pain than vice-versa.^38^ Dysmenorrhea and heavy bleeding can directly reduce sleep quality, and hormonal fluctuations over the menstrual cycle can also affect sleep quality as estrogen and progesterone are involved in sleep regulation through receptors in the central nervous system.^37^ In turn, poor sleep may increase dysmenorrhea and menstrual bleeding through the activation of prostaglandins, and through disruption of circadian rhythms which may cause inconsistent regulation of menstrual-related hormones.^14^ There are few studies on dysmenorrhea and sleep among adolescents in African countries. One cross-sectional survey among 4122 adolescents and young women aged 12-25 years in Egypt found strong evidence of an association between dysmenorrhea and insomnia (aOR = 2.6, 95%CI 1.6-4.3),^39^ and another study found a small and nonsignificant association.^40^

Participants who reported using only reusable products at LMP were more likely to report poor sleep quality and tiredness than those who used disposable pads. Reusable menstrual pads are cheaper and more environmentally friendly than disposable pads but come with challenges such as washing and drying appropriately. This takes time and relies on an appropriate social and physical environment with privacy, water, soap, buckets, and drying facilities.^41^

Poor sleep quality and tiredness were both strongly associated with reported experiences of menstrual teasing, unmet menstrual practice needs, and poorer menstrual self-efficacy. To our knowledge, this is the first study to look at associations between sleep and these central aspects of menstrual health^23^ and suggest that improving confidence to manage menstruation is important for good sleep.

### Association of sleep with mental health problems

The association that we found between poor sleep and poor mental health supports findings from other countries and ours is the first longitudinal study to assess the relationship between sleep and mental health among adolescents in Africa.^18^ This association is bidirectional,^16–18^ with biological, psychological, and social mechanisms interacting to increase vulnerability to both sleep problems and internalizing disorders.^19^ A cross-sectional study among 378 adolescents in Nigeria found that poor sleep (PSQI > =5) was associated with anxiety (aOR = 1.20, 95%CI 1.10-1.32) and depression (aOR = 1.12, 95%CI 1.00-1.25),^42^ and a second study from Nigeria also found an association between poor sleep quality and depression.^43^ Our study confirms this association and shows that poor sleep precedes poor mental health.

### Association of sleep with examination performance

Poor sleep quality at both baseline and endline was associated with a poorer endline examination performance, and there was some evidence of a trend between the proportion of nights with poor sleep and endline examination performance. To our knowledge, these are the first data showing this association in Africa and may help change perceptions about the importance of sleep quality for educational performance.

## Strengths and limitations

The study was nested in a randomized controlled trial of a menstrual health intervention on mental health and educational outcomes. A strength of our study is the use of baseline and endline surveys 1 year apart, and a longitudinal diary substudy with daily data collected over 16 weeks, followed by the endline survey. This allows us to disentangle temporal effects to some extent. However, the study was not designed to investigate sleep health in detail and key limitations are that our data are restricted to girls and that we only had questions about subjective sleep quality and tiredness rather than other dimensions of sleep health in the Buysse framework (alertness, timing, efficiency, and duration),^44^ and that we did not use validated tools for assessing sleep quality, duration, or insomnia. Sleep epidemiology in boys in Uganda is likely to share some socio-ecological determinants with that of girls, but there are differences in adolescent sleep neurophysiology by gender after puberty^45^ and socio-cultural differences in behaviors.^46^

Our detailed assessment of socio-demographic, menstrual, and mental health factors allowed us to adjust these factors for a range of potential confounders according to our conceptual frameworks. However, sleep health is affected by a broad range of societal, social, and individual factors, and we did not collect data on other potentially relevant factors at the societal level (environmental factors, e.g., noise, light, temperature, urban/rural status), social level (family dynamics including adverse child experiences, parental occupation, the social and physical school environment including school start/end time, study time, social networks), and individual level (dys-functional beliefs and attitudes about sleep, social media use, caffeine use, chronotype, social jetlag), which may have contributed to residual confounding.

Rather than using standard measures of sleep health, we had two single item questions from the CHU9-D. This may not be a limitation, as a recent review questioned the applicability of sleep health tools such as the PSQI in LMICs as these have only been validated in high-income countries,^47^ and cutpoints for symptom-based disorders like insomnia may require culturally specific and sensitive approaches. We found one study validating the PSQI among youth in Africa (among university students in Nigeria) that found it to be of moderate use (AUC of 0.69, below the recommended value of 0.80), with low specificity (54.5%).^48^ Further work is needed to validate sleep health questionnaires in the African region and for adolescents, including the use of objective measures such as actigraphy and smartphone-based sensors.

## Conclusion

Sleep problems are prevalent among in-school female adolescents in Uganda, and there is strong evidence for associations with poor menstrual health and mental health, and to a lesser extent with educational performance. Further work is needed to better understand the social and physical environment around sleep in this setting. Our findings suggest that improving sleep may influence menstrual health, mental health, and educational performance. Further work is needed to modify evidence-based sleep health interventions for this setting, including the potential to implement and evaluate school-level interventions such as later school start times and the provision of group cognitive behavior therapy for insomnia for those who need it to improve sleep.

## Supplementary Material

Supplementary data associated with this article can be found in the online version at doi:10.1016/j.sleh.2024.12.007.

Supplementary material

## Figures and Tables

**Table 1:** Factors associated with sleep problems and feeling tired at baseline

	Total	Sleep problems N (%)	Adjusted OR^a^ (95%CI)	*p*-value	Feeling tired N (%)	Adjusted OR^a^ (95%CI)	*p*-value
N	3841	580 (15.1%)			829 (21.6%)		
Level 1: Sociodemographic variables							
*Societal level factors*							
District							
Kalungu	859	117 (13.6%)	1	.04	161 (18.7%)	1	.10
Wakiso	2982	463 (15.5%)	1.29 (1.01-1.65)		668 (22.4%)	1.19 (0.97-1.47)	
*Social-level factors*							
School ownership							
Government	1348	205 (15.2%)	1	.92	270 (20.0%)	1	.41
Private	2493	375 (15.0%)	0.99 (0.79-1.24)		559 (22.4%)	1.09 (0.89-1.33)	
UNEB score at baseline							
Low UNEB	1817	273 (15.0%)	1	.45	382 (21.0%)	1	.57
High UNEB	2024	307 (15.2%)	1.08 (0.89-1.31)		447 (22.1%)	1.05 (0.88-1.25)	
Proportion of participants who are boarding							
Less than 50%	2140	322 (15.0%)	1	.81	425 (19.9%)	1	.91
Greater or equal to 50%	1701	258 (15.2%)	1.03 (0.82-1.28)		404 (23.8%)	1.01 (0.83-1.23)	
Number of female participants in the study							
Fewer than 59	1254	203 (16.2%)	1	.07	236 (18.8%)	1	.02
Greater or equal to 59	2587	377 (14.6%)	0.83 (0.68-1.01)		593 (22.9%)	1.23 (1.03-1.48)	
Religion							
Catholic	1219	183 (15.0%)	1	.47	232 (19.0%)	1	.002
Protestant/Born Again/SDA	1491	218 (14.6%)	0.91 (0.73-1.14)		307 (20.6%)	1.02 (0.84-1.24)	
Muslim	1114	177 (15.9%)	1.07 (0.85-1.35)		287 (25.8%)	1.40 (1.14-1.72)	
None/Other	17	2 (11.8%)	0.56 (0.12-2.54)		3 (17.6%)	0.79 (0.22-2.82)	
Ethnicity							
Muganda	2637	379 (14.4%)	1	.07	527 (20.0%)	1	.001
Non Muganda	1204	201 (16.7%)	1.20 (0.99-1.46)		302 (25.1%)	1.33 (1.13-1.58)	
Day/boarding status							
Day	2124	319 (15.0%)	1	.41	412 (19.4%)	1	.01
Boarding	1717	261 (15.2%)	1.09 (0.88-1.35)		417 (24.3%)	1.28 (1.06-1.54)	
Primary caregiver							
Mother	2258	323 (14.3%)	1	.05	471 (20.9%)	1	.36
Father	933	142 (15.2%)	1.19 (0.96-1.49)		213 (22.8%)	1.12 (0.92-1.35)	
Other	650	115 (17.7%)	1.32 (1.04-1.68)		145 (22.3%)	1.14 (0.91-1.41)	
Household size							
> =8	1419	198 (14.0%)	1	.03^c^	308 (21.7%)	1	.23
6-7	1243	187 (15.0%)	1.16 (0.93-1.44)		287 (23.1%)	1.09 (0.91-1.32)	
0-5	1179	195 (16.5%)	1.29 (1.03-1.60)		234 (19.8%)	0.92 (0.76-1.12)	
Socioeconomic status							
Highest	771	157 (20.4%)	1	.003^c^	177 (23.0%)	1	.15^c^
Medium-high	784	118 (15.1%)	1.03 (0.76-1.38)		172 (21.9%)	0.95 (0.75-1.22)	
Medium	757	91 (12.0%)	0.86 (0.63-1.18)		139 (18.4%)	0.84 (0.65-1.08)	
Medium-low	770	107 (13.9%)	1.13 (0.83-1.52)		162 (21.0%)	1.10 (0.85-1.42)	
Lowest	759	107 (14.1%)	1.58 (1.17-2.12)		179 (23.6%)	1.16 (0.89-1.50)	
Number of meals eaten on the previous day							
Three or more	1207	142 (11.8%)	1	< .001^c^	228 (18.9%)	1	.02^c^
Two	1948	281 (14.4%)	1.20 (0.97-1.50)		437 (22.4%)	1.23 (1.02-1.48)	
One or fewer	686	157 (22.9%)	2.09 (1.61-2.70)		164 (23.9%)	1.32 (1.05-1.67)	
*Individual-level factors*							
Age group (y)							
< 15	376	59 (15.7%)	1	.51	98 (26.1%)	1	.008
15	1543	211 (13.7%)	0.84 (0.61-1.17)		306 (19.8%)	0.72 (0.55-0.95)	
16	1391	229 (16.5%)	1.01 (0.73-1.40)		330 (23.7%)	0.94 (0.71-1.24)	
17	408	60 (14.7%)	0.89 (0.59-1.34)		75 (18.4%)	0.71 (0.49-1.01)	
18+	123	21 (17.1%)	1.03 (0.59-1.82)		20 (16.3%)	0.61 (0.35-1.05)	
Started menstrual periods?							
Yes	3705	558 (15.1%)	1	.13	796 (21.5%)	1	.90
No	106	14 (13.2%)	0.82 (0.46-1.48)		26 (24.5%)	1.10 (0.70-1.75)	
Don’t know	30	8 (26.7%)	2.25 (0.98-5.16)		7 (23.3%)	1.08 (0.45-2.56)	
Level 2: Menstrual management at LMP (among 3281 girls who had menstruated in the past 6 mo)							
Pain management in LMP^b^							
No pain	855	81 (9.5%)	1	< .001	141 (16.5%)	1	< .001^c^
Effective pain management	1517	239 (15.8%)	1.71 (1.30-2.25)		329 (21.7%)	1.43 (1.14-1.79)	
No effective pain management	909	149 (16.4%)	1.74 (1.29-2.33)		240 (26.4%)	1.78 (1.40-2.26)	
Pain relief at LMP^b^							
No pain	855	81 (9.5%)	1	< .001^c^	141 (16.5%)	1	< .001
All/most	973	146 (15.0%)	1.62 (1.21-2.18)		216 (22.2%)	1.46 (1.15-1.86)	
None/some	1453	242 (16.7%)	1.78 (1.36-2.35)		353 (24.3%)	1.63 (1.30-2.04)	
Menstrual product used^b^							
Disposable only	2214	266 (12.0%)	1		445 (20.1%)	1	
Reusable only	530	130 (24.5%)	2.23 (1.73-2.87)	< .001	138 (26.0%)	1.48 (1.17-1.87)	.001
Both reusable and disposable	533	72 (13.5%)	1.10 (0.83-1.47)		126 (23.6%)	1.32 (1.04-1.66)	
Level 3: Menstrual confidence and experience at LMP							
Experience teasing about period by boys or girls^b^							
No	2815	357 (12.7%)	1	.001	569 (20.2%)	1	.001
Yes	466	112 (24.0%)	1.57 (1.21-2.04)		141 (30.3%)	1.50 (1.19-1.58)	
MPNS tertile^b^							
High	1105	84 (7.6%)	1	< .001^c^	172 (15.6%)	1	< .001^c^
Medium	1079	138 (12.8%)	1.59 (1.18, 2.14)		212 (19.6%)	1.25 (0.99-1.58)	
Low	1068	243 (22.8%)	2.68 (1.99, 3.60)		322 (30.1%)	1.98 (1.56-2.51)	
SAMNS tertile^b^							
High	1115	115 (10.3%)	1	.02^c^	183 (16.4%)	1	< .001^c^
Medium	1098	154 (14.0%)	1.23 (0.94, 1.61)		243 (22.1%)	1.33 (1.06-1.66)	
Low	1068	200 (18.7%)	1.40 (1.07, 1.83)		284 (26.6%)	1.52 (1.21-1.91)	
Level 4: Mental health problems and educational performance Anxious about next period^b^							
No	1826	179 (9.8%)	1	< .001	294 (16.1%)	1	< .001
Yes	1455	290 (19.9%)	1.62 (1.28-2.06)		416 (28.6%)	1.56 (1.28-1.90)	
SDQ score							
0-15 (Normal)	2803	294 (10.5%)	1	< .001^c^	473 (16.9%)	1	< .001
16-19 (Borderline)	626	145 (23.2%)	1.75 (1.35-2.27)		206 (32.9%)	1.93 (1.54-2.43)	
20-40 (High)	412	141 (34.2%)	2.40 (1.80-3.19)		150 (36.4%)	1.92 (1.47-2.52)	
UNEB exam score tertile							
High	1120	124 (11.1%)	1	.06	228 (20.4%)	1	.07
Medium	1158	177 (15.3%)	1.33 (1.02, 1.73)		225 (19.4%)	0.92 (0.74, 1.15)	
Low	1141	203 (17.8%)	1.36 (1.03, 1.79)		276 (24.2%)	1.19 (0.94, 1.49)	

Abbreviations: LMP, last menstrual period; MPNS, Menstrual Practice Needs Scale; SAMNS, Self-efficacy in Addressing Menstrual Needs Scale; SDQ, Strength and Difficulties Questionnaire; UNEB, Uganda National Examinations Board.

a Adjusted for other variables at the same or a more distal level.

b Among participants with a last menstrual period within the past 6 mo.

c *p*-value for trend.

**Table 2a:** Association of sleep problems (exposure) with menstrual health, mental health and examination performance outcomes at endline

Outcome (at endline)	No sleep problems	Sleep problems at baseline	Sleep problems at endline	Sleep problems at both time points	*p*-value
Total (N = 2901)	2185	309	297	110	
MPNS score (mean; SD)^a,b^	2.39 (0.45)	2.21 (0.49)	2.04 (0.57)	1.87 (0.57)	
aMD (95%CI)	0	−0.04 (−0.09, 0.01)	−0.27 (−0.32, −0.22)	−0.33 (−0.41, −0.24)	< .001
aSMD (95%CI)	0	−0.08 (−0.19, 0.03)	−0.55 (−0.66, −0.44)	−0.66 (−0.84, −0.48)	
SAMNS score (mean; SD)^a,b^	67.68 (18.06)	63.90 (18.28)	60.73 (19.84)	55.10 (20.77)	
aMD (95%CI)	0	−0.89 (−3.05, 1.27)	−4.92 (−7.07, −2.77)	−8.84 (−12.36, −5.31)	< .001
aSMD (95%CI)	0	−0.05 (−0.16, 0.07)	−0.26 (−0.38, −0.15)	−0.48 (−0.67, −0.29)	
SDQ score (mean; SD)^b^	9.62 (4.98)	12.08 (5.18)	14.21 (5.76)	16.25 (5.68)	
aMD (95%CI)	0	0.73 (0.15, 1.31)	3.45 (2.87, 4.03)	4.23 (3.30, 5.16)	< .001
aSMD (95%CI)	0	0.13 (0.03, 0.24)	0.63 (0.52, 0.73)	0.77 (0.60, 0.94)	
UNEB score (mean; SD)^b^	0.07 (1.00)	−0.20 (0.95)	−0.02 (0.95)	−0.36 (1.08)	
aSMD (95%CI)	0	−0.12 (−0.23, −0.01)	−0.07 (−0.18, 0.04)	−0.23 (−0.42, −0.05)	.02

Abbreviations: aMD, adjusted mean differences; aSMD, adjusted standardized mean differences; MPNS, Menstrual Practice Needs Scale; SAMNS, Self-efficacy in Addressing Menstrual Needs Scale; SDQ, Strength and Difficulties Questionnaire; UNEB, Uganda National Examinations Board.

a Among participants with a last menstrual period within the past 6 mo.

b Adjusted for factors shown in Table 1, and trial arm.

**Table 2b:** Association of feeling tired (exposure) with mental health and menstrual health at endline (outcome)

Outcome	Not tired	Felt tired at baseline	Felt tired at endline	Felt tired at both time points	*p*-value
Total (N = 2901)	1769	351	535	246	
MPNS score (mean; SD)^a,b^	2.39 (0.47)	2.26 (0.48)	2.21 (0.50)	2.06 (0.55)	
aMD (95%CI)	0	−0.04 (−0.09, 0.01)	−0.12 (−0.17, −0.08)	−0.22 (−0.28, −0.16)	< .001
aSMD (95%CI)	0	−0.08 (−0.19, 0.02)	−0.25 (−0.34, −0.16)	−0.44 (−0.56, −0.31)	
SAMNS score (mean; SD)^a,b^	68.20 (18.31)	64.94 (18.23)	63.32 (18.30)	58.42 (19.43)	
aMD (95%CI)	0	−1.12 (−3.18, 0.94)	−2.96 (−4.70, −1.22)	−5.95 (−8.43, −3.48)	< .001
aSMD (95%CI)	0	−0.06 (−0.17, 0.05)	−0.16 (−0.25, −0.07)	−0.32 (−0.45, −0.19)	
SDQ score (mean; SD)^b^	9.43 (5.04)	11.44 (5.65)	12.30 (5.42)	14.07 (5.33)	
aMD (95%CI)	0	0.66 (0.10, 1.22)	1.91 (1.43, 2.38)	2.82 (2.15, 3.49)	< .001
aSMD (95%CI)	0	0.12 (0.02, 0.22)	0.35 (0.26, 0.43)	0.51 (0.39, 0.63)	
UNEB score (mean; SD)^b^	0.03 (1.01)	−0.11 (0.96)	0.07 (0.96)	0.01 (1.08)	
aSMD (95%CI)	0	−0.07 (−0.17, 0.04)	0.02 (−0.07, 0.11)	−0.03 (−0.16, 0.10)	.51

Abbreviations: aMD, adjusted mean differences; aSMD, adjusted standardized mean differences; MPNS, Menstrual Practice Needs Scale; SAMNS, Self-efficacy in Addressing Menstrual Needs Scale; SDQ, Strength and Difficulties Questionnaire; UNEB, Uganda National Examinations Board.

a Among participants with a last menstrual period within the past 6 mo.

b Adjusted for factors shown in Table 1, and trial arm.

**Table 3:** Associations of proportion of nights with poor sleep with subsequent menstrual health, mental health and examination performance

Outcome (at endline)	Proportion of nights with poor sleep prior to endline (median 9 wk)				*p*-value for trend
	None	1%-9%	10%-19%	> =20%	
Total number	354 (26.7%)	648 (48.8%)	205 (15.4%)	121 (9.1%)	
Tired (%)	15.6%	22.0%	32.3%	44.5%	
aOR (95%CI)	1	1.43 (1.00, 2.05)	2.33 (1.51, 3.60)	3.52 (2.17, 5.72)	< .001
MPNS score (mean; SD)	2.44 (0.45)	2.39 (0.45)	2.27 (0.52)	2.17 (0.54)	
aMD (95%CI)^a^	0	-0.02 (-0.07, 0.04)	−0.13 (−0.21, −0.06)	−0.19 (−0.28, −0.10)	< .001
aSMD (95%CI)^b^	0	−0.04 (−0.16, 0.08)	−0.28 (−0.44, −0.13)	−0.39 (−0.58, −0.21)	
SAMNS score (mean; SD)	68.91 (18.10)	67.48 (18.01)	64.91 (17.52)	63.98 (20.66)	
aMD (95%CI)^a^	0	−0.12 (−2.38, 2.14)	−2.90 (−5.92, 0.13)	−3.80 (−7.46, −0.14)	.01
aSMD (95%CI)^b^	0	−0.01 (−0.13, 0.12)	−0.16 (−0.32, 0.01)	−0.21 (−0.41, −0.01)	
SDQ score (mean; SD)	8.84 (4.87)	9.71 (5.07)	11.68 (5.44)	13.02 (6.04)	
aMD (95%CI)^a^	0	0.30 (−0.31, 0.91)	1.71 (0.89, 2.53)	2.66 (1.68, 3.64)	< .001
aSMD (95%CI)^b^	0	0.06 (−0.06, 0.17)	0.32 (0.17, 0.47)	0.50 (0.32, 0.68)	
UNEB z-score (mean; SD)	−0.01 (1.0)	0.02 (1.00)	−0.02 (0.92)	−0.10 (1.14)	
aSMD (95%CI)^a^	0	−0.02 (−0.14, 0.09)	−0.09 (−0.24, 0.06)	−0.17 (−0.37, 0.02)	.05

Abbreviations: aMD, adjusted mean differences; aSMD, adjusted standardized mean differences; MPNS, Menstrual Practice Needs Scale; SAMNS, Self-efficacy in Addressing Menstrual Needs Scale; SDQ, Strength and Difficulties Questionnaire; UNEB, Uganda National Examinations Board.

a Adjusted for level 1 to level 4 variables in Fig. 1, the baseline of the outcome variable and school-level clustering.

b As above, but estimated from standardized outcome variables.

**Table 4:** Associations of menstrual and educational factors (exposures) with poor sleep (outcome) using daily diary data

Characteristic	Total number of days	Proportion with poor sleep	Unadjusted OR (95%CI)	OR (95%CI) adjusted for baseline confounders	OR (95%CI) adjusted for baseline confounders and other diary data^a^
All days	144,884	6.9%			
Menstruation					
No	124,487	5.4%	1	1	
Yes	19,541	16.4%	4.34 (3.70−5.08)	4.32 (3.69−5.06)	n/a
Menstrual flow					*p* < .001
Not menstruating	124,487	5.4%	1	1	1
Light	9352	11.0%	2.52 (2.15−2.95)	2.51 (2.14−2.94)	1.14 (0.98−1.32)
Medium	7065	14.4%	4.05 (3.38−4.85)	4.03 (3.36−4.83)	1.58 (1.26−1.98)
Heavy	3124	36.6%	13.57 (10.73−17.16)	13.53 (10.68−17.12)	3.96 (3.11−5.04)
Period pain (on period days^b^)					*p* < .001
No	15,589	5.9%	1	1	1
Yes	8556	30.5%	7.83 (6.69−9.17)	7.68 (6.56−9.00)	5.79 (4.71−7.11)
School open					*p* < .001
Yes	85,874	6.6%	1	1	1
No	57,906	7.4%	1.22 (1.13−1.33)	1.22 (1.13−1.32)	1.25 (1.15−1.36)
Exam day if school open					*p* = .08
No	72,696	6.7%	1	1	1
Yes	12,368	6.2%	0.91 (0.80−1.03)	0.91 (0.81−1.04)	0.89 (0.79−1.01)
Attended all classes if school open					*p* < .001
Yes	73,153	5.7%	1	1	1
No	12,721	12.5%	2.44 (2.10−2.84)	2.44 (2.10−2.84)	2.22 (1.89−2.60)

a Menstrual flow, period pain and exam day are adjusted for each other (restricted to days on which school was open) but not for attended all classes which are likely to be on the causal pathway from these variables. Attended all classes is adjusted for menstrual flow, period pain and exam day. School open was adjusted for menstrual flow and period pain.

b Days menstruating plus day prior to menses.

## Data Availability

The data are available on request from LSHTM Data Compass.
