# Supplementary material for: Sleep health epidemiology and associations with menstrual health, mental health, and educational performance among in-school female adolescents in Uganda: A longitudinal study
Source: Sleep Health. Author manuscript; Available in PMC 2026 Apr 7. (PMC7618985; doi:10.1016/j.sleh.2024.12.007)
Supplement: Supplementary material [file EMS213001-supplement-Supplementary_material.docx]

| **Table A1: Factors associated with sleep problems & feeling tired at baseline** | | | | | | | | | | |
| --- | --- | --- | --- | --- | --- | --- | --- | --- | --- | --- |
|  | **Total** | **Sleep problems**  **N (%)** | **Adjusted OR^a^ (95%CI)** | | **p-value** | | **Feeling tired**  **N (%)** | | **Adjusted OR^a^ (95%CI)** | **p-value** |
| **N** | 3841 | 297 (7.7%) |  | |  | | 441 (11.5%) | |  |  |
| **Level 1: Sociodemographic variables** | | | |  | |  | |  | | |
| **Societal level factors** |  |  |  | |  | |  | |  |  |
| **District** |  |  |  | |  | |  | |  |  |
| Kalungu | 859 | 54 (6.3%) | 1 | | 0.07 | | 93 (10.8%) | | 1 | 0.99 |
| Wakiso | 2982 | 243 (8.2%) | 1.38 (0.98-1.96) | |  | | 348 (11.7%) | | 1.00 (0.77-1.31) |  |
| **Social-level factors** |  |  |  | |  | |  | |  |  |
| **School ownership** |  |  |  | |  | |  | |  |  |
| Government | 1348 | 102 (7.6%) | 1 | | 0.85 | | 147 (10.9%) | | 1 | 0.31 |
| Private | 2493 | 195 (7.8%) | 0.97 (0.70-1.33) | |  | | 294 (11.8%) | | 1.14 (0.88-1.48) |  |
| **UNEB score at baseline** |  |  |  | |  | |  | |  |  |
| Low UNEB | 1817 | 133 (7.3%) | 1 | | 0.26 | | 208 (11.4%) | | 1 | 0.80 |
| High UNEB | 2024 | 164 (8.1%) | 1.17 (0.89-1.55) | |  | | 233 (11.5%) | | 1.03 (0.83-1.28) |  |
| **Proportion of participants who are boarding** |  |  |  | |  | |  | |  |  |
| Less than 50% | 2,140 | 159 (7.4%) | 1 | | 0.30 | | 238 (11.1%) | | 1 | 0.57 |
| Greater or equal to 50% | 1,701 | 138 (8.1%) | 1.18 (0.87-1.60) | |  | | 203 (11.9%) | | 0.93 (0.73-1.19) |  |
| **Number of female participants in the study** |  |  |  | |  | |  | |  |  |
| Fewer than 59 | 1,254 | 100 (8.0%) | 1 | | 0.38 | | 128 (10.2%) | | 1 | 0.09 |
| Greater or equal to 59 | 2,587 | 197 (7.6%) | 0.88 (0.67-1.17) | |  | | 313 (12.1%) | | 1.22 (0.97-1.54) |  |
| **Religion** |  |  |  | |  | |  | |  |  |
| Catholic | 1219 | 93 (7.6%) | 1 | | 0.81 | | 119 (9.8%) | | 1 | 0.08 |
| Protestant/Born Again/SDA | 1491 | 111 (7.4%) | 0.91 (0.67-1.22) | |  | | 171 (11.5%) | | 1.14 (0.89-1.48) |  |
| Muslim | 1114 | 91 (8.2%) | 1.03 (0.75-1.42) | |  | | 149 (13.4%) | | 1.42 (1.09-1.84) |  |
| None/Other | 17 | 2 (11.8%) | 1.28 (0.28-5.90) | |  | | 2 (11.8%) | | 1.04 (0.23-4.69) |  |
| **Ethnicity** |  |  |  | |  | |  | |  |  |
| Muganda | 2637 | 196 (7.4%) | 1 | | 0.43 | | 279 (10.6%) | | 1 | 0.02 |
| Non Muganda | 1204 | 101 (8.4%) | 1.11 (0.86-1.44) | |  | | 162 (13.5%) | | 1.31 (1.05-1.62) |  |
| **Day/boarding status** |  |  |  | |  | |  | |  |  |
| Day | 2124 | 166 (7.8%) | 1 | | 0.75 | | 232 (10.9%) | | 1 | 0.42 |
| Boarding | 1717 | 131 (7.6%) | 0.96 (0.72-1.27) | |  | | 209 (12.2%) | | 1.10 (0.87-1.40) |  |
| **Primary caregiver** |  |  |  | |  | |  | |  |  |
| Mother | 2258 | 172 (7.6%) | 1 | | 0.75 | | 256 (11.3%) | | 1 | 0.57 |
| Father | 933 | 68 (7.3%) | 1.04 (0.77-1.40) | |  | | 115 (12.3%) | | 1.12 (0.88-1.42) |  |
| Other | 650 | 57 (8.8%) | 1.13 (0.82-1.57) | |  | | 70 (10.8%) | | 0.96 (0.72-1.27) |  |
| **Household size** |  |  |  | |  | |  | |  |  |
| >=8 | 1419 | 103 (7.3%) | 1 | | 0.59 | | 158 (11.1%) | | 1 | 0.62 |
| 6-7 | 1243 | 97 (7.8%) | 1.11 (0.83-1.50) | |  | | 152 (12.2%) | | 1.12 (0.88-1.42) |  |
| 0-5 | 1179 | 97 (8.2%) | 1.16 (0.86-1.56) | |  | | 131 (11.1%) | | 1.02 (0.79-1.31) |  |
| **Socioeconomic status** |  |  |  | |  | |  | |  |  |
| Highest | 771 | 79 (10.2%) | 1 | | 0.06 | | 101 (13.1%) | | 1 | 0.54 |
| Medium-high | 784 | 55 (7.0%) | 0.91 (0.62-1.33) | |  | | 88 (11.2%) | | 1.03 (0.75-1.41) |  |
| Medium | 757 | 44 (5.8%) | 0.69 (0.46-1.04) | |  | | 75 (9.9%) | | 0.89 (0.64-1.24) |  |
| Medium-low | 770 | 56 (7.3%) | 0.84 (0.56-1.25) | |  | | 88 (11.4%) | | 1.03 (0.74-1.43) |  |
| Lowest | 759 | 63 (8.3%) | 1.22 (0.83-1.79) | |  | | 89 (11.7%) | | 1.19 (0.85-1.66) |  |
| **Number of meals eaten on the previous day** |  |  |  | |  | |  | |  |  |
| Three or more | 1207 | 70 (5.8%) | 1 | | <0.001^c^ | | 113 (9.4%) | | 1 | <0.001^c^ |
| Two | 1948 | 134 (6.9%) | 1.18 (0.87-1.60) | |  | | 227 (11.7%) | | 1.26 (0.99-1.61) |  |
| One or fewer | 686 | 93 (13.6%) | 2.47 (1.77-3.46) | |  | | 101 (14.7%) | | 1.65 (1.23-2.21) |  |
| **Individual-level factors** |  |  |  | |  | |  | |  |  |
| **Age group (years)** |  |  |  | |  | |  | |  |  |
| <15 | 376 | 34 (9.0%) | 1 | | 0.37 | | 50 (13.3%) | | 1 | 0.09 |
| 15 | 1543 | 106 (6.9%) | 0.77 (0.51-1.16) | |  | | 158 (10.2%) | | 0.78 (0.55-1.10) |  |
| 16 | 1391 | 112 (8.1%) | 0.91 (0.59-1.38) | |  | | 182 (13.1%) | | 1.04 (0.73-1.48) |  |
| 17 | 408 | 31 (7.6%) | 0.90 (0.53-1.53) | |  | | 39 (9.6%) | | 0.76 (0.48-1.21) |  |
| 18+ | 123 | 14 (11.4%) | 1.32 (0.67-2.63) | |  | | 12 (9.8%) | | 0.77 (0.39-1.54) |  |
| **Started menstrual periods?** |  |  |  | |  | |  | |  |  |
| Yes | 3705 | 286 (7.7%) | 1 | | 0.59 | | 419 (11.3%) | | 1 | 0.27 |
| No | 106 | 10 (9.4%) | 1.21 (0.61-2.40) | |  | | 18 (17.0%) | | 1.54 (0.91-2.62) |  |
| Don't know | 30 | 1 (3.3%) | 0.41 (0.06-3.10) | |  | | 4 (13.3%) | | 1.21 (0.42-3.53) |  |
| **Level 2: Menstrual management at LMP (among 3281 girls who had menstruated in the past 6 months)** | | | | | | | | | | |
| **Pain management in LMP^b^** |  |  |  | |  | |  | |  |  |
| No pain | 855 | 39 (4.6%) | 1 | | 0.008^c^ | | 70 (8.2%) | | 1 | <0.001 ^c^ |
| Effective pain management | 1517 | 126 (8.3%) | 1.78 (1.22-2.60) | |  | | 163 (10.7%) | | 1.32 (0.98-1.78) |  |
| No effective pain mngment | 909 | 78 (8.6%) | 1.80 (1.20-2.71) | |  | | 132 (14.5%) | | 1.83 (1.34-2.50) |  |
| **Pain relief at LMP^b^** |  |  |  | |  | |  | |  |  |
| No pain | 855 | 39 (4.6%) | 1 | | 0.006 | | 70 (8.2%) | | 1 | 0.02 |
| None/some | 973 | 81 (8.3%) | 1.81 (1.21-2.71) | |  | | 112 (11.5%) | | 1.43 (1.04-1.97) |  |
| All/most | 1453 | 123 (8.5%) | 1.78 (1.22-2.61) | |  | | 183 (12.6%) | | 1.56 (1.16-2.10) |  |
| **Menstrual product used^b^** |  |  |  | |  | |  | |  |  |
| Disposable only | 2214 | 133 (6.0%) | 1 | | <0.001 | | 216 (9.8%) | | 1 | 0.001 |
| Reusable only | 530 | 71 (13.4%) | 2.29 (1.64-3.18) | |  | | 81 (15.3%) | | 1.70 (1.26-2.27) |  |
| Both reusable & disposable | 533 | 38 (7.1%) | 1.20 (0.82-1.76) | |  | | 67 (12.6%) | | 1.37 (1.02-1.85) |  |
| **Level 3: Menstrual confidence and experience at LMP** | | | | | | | | | |  |
| **Experience teasing about period by boys or girls^b^** |  |  |  | |  | |  | |  |  |
| No | 2815 | 180 (6.4%) | 1 | | 0.001 | | 282 (10.0%) | | 1 | 0.001 |
| Yes | 466 | 63 (13.5%) | 1.72 (1.24-2.39) | |  | | 83 (17.8%) | | 1.65 (1.24-2.18) |  |
| **MPNS tertile^b^** |  |  |  | |  | |  | |  |  |
| High | 1105 | 46 (4.2%) | 1 | | <0.001^c^ | | 81 (7.3%) | | 1 | <0.001^c^ |
| Medium | 1079 | 65 (6.0%) | 1.31 (0.88, 1.96) | |  | | 104 (9.6%) | | 1.23 (0.90-1.69) |  |
| Low | 1068 | 129 (12.1%) | 2.26 (1.53, 3.34) | |  | | 177 (16.6%) | | 1.97 (1.44-2.69) |  |
| **SAMNS tertile^b^** |  |  |  | |  | |  | |  |  |
| High | 1115 | 66 (5.9%) | 1 | | 0.18 | | 90 (8.1%) | | 1 | 0.03^c^ |
| Medium | 1098 | 71 (6.5%) | 0.97 (0.67, 1.39) | |  | | 127 (11.6%) | | 1.33 (0.99-1.78) |  |
| Low | 1068 | 106 (9.9%) | 1.26 (0.89, 1.78) | |  | | 148 (13.9%) | | 1.41 (1.05-1.90) |  |
| **Level 4: Mental health problems and educational performance** | | | | | | | | |  |  |
| **Anxious about next period^b^** |  |  |  | |  | |  | |  |  |
| No | 1826 | 80 (4.4%) | 1 | | <0.001 | | 159 (8.7%) | | 1 | 0.05 |
| Yes | 1455 | 163 (11.2%) | 2.13 (1.53-2.97) | |  | | 206 (14.2%) | | 1.31 (1.01-1.70) |  |
| **SDQ score** |  |  |  | |  | |  | |  |  |
| 0-15 (Normal) | 2803 | 114 (4.7%) | 1 | | <0.001^c^ | | 232 (8.3%) | | 1 | <0.001^c^ |
| 16-19 (Borderline) | 626 | 60 (11.5%) | 1.82 (1.25-2.66) | |  | | 114 (18.2%) | | 1.63 (1.20-2.23) |  |
| 20-40 (High) | 412 | 69 (20.5%) | 2.73 (1.83-4.08) | |  | | 95 (23.1%) | | 1.95 (1.37-2.77) |  |
| **UNEB exam score** |  |  |  | |  | |  | |  |  |
| High | 1120 | 58 (5.7%) | 1 | | 0.06^c^ | | 106 (9.5%) | | 1 | 0.24 |
| Medium | 1158 | 72 (7.2%) | 1.16 (0.79, 1.71) | |  | | 120 (10.4%) | | 1.05 (0.77, 1.43) |  |
| Low | 1141 | 79 (8.7%) | 1.31 (0.88, 1.95) | |  | | 158 (13.8%) | | 1.29 (0.94, 1.77) |  |

^a^ Adjusted for other variables at the same or a more distal level

^b^ Among participants with a last menstrual period within the past 6 months

^c^ p-value for trend

**Table A2a: Association of sleep problems (exposure) with menstrual health, mental health and examination performance outcomes at endline**

| **Outcome (at endline)** | **No sleep problems** | **Sleep problems at baseline** | **Sleep problems at endline** | **Sleep problems at both time points** | **p-value** |
| --- | --- | --- | --- | --- | --- |
| **Total (N=2901)** | **2541** | **166** | **155** | **39** |  |
|  |  |  |  |  |  |
| **MPNS score (mean; SD)^[[1]](#footnote-2)^** | 2.36 (0.47) | 2.17 (0.51) | 1.92 (0.57) | 1.87 (0.53) |  |
| aMD (95%CI)^b^ | 0 | -0.02 (-0.09, 0.05) | -0.31 (-0.39, -0.24) | -0.28 (-0.43, -0.13) | <0.001 |
| aSMD (95%CI)^b^ | 0 | -0.03 (-0.18, 0.11) | -0.63 (-0.78, -0.49) | -0.57 (-0.86, -0.27) |  |
|  |  |  |  |  |  |
| **SAMNS score (mean; SD)^a^** | 66.96 (18.25) | 62.52 (19.25) | 58.06 (20.43) | 56.16 (22.13) |  |
| aMD (95%CI)^b^ | 0 | -1.17 (-3.98, 1.65) | -5.96 (-8.91, -3.02) | -7.11 (-12.95, -1.27) | <0.001 |
| aSMD (95%CI)^b^ | 0 | -0.06 (-0.21, 0.09) | -0.32 (-0.48, -0.16) | -0.39 (-0.70, -0.07) |  |
|  |  |  |  |  |  |
| **SDQ score (mean; SD)** | 10.03 (5.10) | 13.04 (5.35) | 15.55 (6.33) | 17.53 (5.63) |  |
| aMD (95%CI)^b^ | 0 | 0.78 (0.01, 1.55) | 3.80 (3.01, 4.59) | 4.91 (3.36, 6.47) | <0.001 |
| aSMD (95%CI)^b^ | 0 | 0.14 (0.00, 0.28) | 0.69 (0.55, 0.83) | 0.89 (0.61, 1.18) |  |
|  |  |  |  |  |  |
| **UNEB score (mean; SD)** | 0.06 (1.00) | -0.30 (0.93) | -0.24 (0.99) | -0.40 (1.08) |  |
| aSMD (95%CI)^[[2]](#footnote-3)^ | 0 | -0.21 (-0.36, -0.06) | -0.21 (-0.37, -0.06) | -0.40 (-0.72, -0.09) | <0.001 |

**Table A2b: Association of feeling tired (exposure) with mental health & menstrual health at endline (outcome)**

| **Outcome** | **Not tired** | **Felt tired at baseline** | **Felt tired at endline** | **Felt tired at both time points** | **p-value** |
| --- | --- | --- | --- | --- | --- |
| **Total (N=2901)** | **2259** | **215** | **340** | **87** |  |
|  |  |  |  |  |  |
| **MPNS score (mean; SD)^a^** | 2.36 (0.48) | 2.18 (0.53) | 2.15 (0.50) | 2.09 (0.51) |  |
| aMD (95%CI)^b^ | 0 | -0.05 (-0.11, 0.01) | -0.13 (-0.18, -0.08) | -0.14 (-0.24, -0.04) | <0.001 |
| aSMD (95%CI)^b^ | 0 | -0.11 (-0.24, 0.02) | -0.26 (-0.37, -0.16) | -0.30 (-0.50, -0.09) |  |
|  |  |  |  |  |  |
| **SAMNS score (mean; SD) ^a^** | 67.13 (18.31) | 62.72 (20.27) | 62.99 (18.43) | 58.75 (20.19) |  |
| aMD (95%CI)^b^ | 0 | -0.97 (-3.50, 1.55) | -1.68 (-3.74, 0.37) | -5.76 (-9.79, -1.73) | <0.001 |
| aSMD (95%CI)^b^ | 0 | -0.05 (-0.19, 0.08) | -0.09 (-0.20, 0.02) | -0.32 (-0.53, -0.10) |  |
|  |  |  |  |  |  |
| **SDQ score (mean; SD)** | 9.84 (5.14) | 12.63 (5.67) | 13.39 (5.74) | 14.36 (5.01) |  |
| aMD (95%CI)^b^ | 0 | 1.22 (0.54, 1.91) | 2.43 (1.87, 2.99) | 2.70 (1.63, 3.77) | <0.001 |
| aSMD (95%CI)^b^ | 0 | 0.22 (0.10, 0.34) | 0.44 (0.34, 0.55) | 0.49 (0.30, 0.68) |  |
|  |  |  |  |  |  |
| **UNEB score (mean; SD)** | 0.04 (0.99) | -0.26 (1.05) | 0.07 (1.02) | 0.09 (0.98) |  |
| aSMD (95%CI)^b^ | 0 | -0.14 (-0.27, -0.01) | 0.02 (-0.09, 0.13) | 0.02 (-0.19, 0.22) | 0.17 |

1. Among participants who had menstruated in the past 6 months [↑](#footnote-ref-2)
2. Adjusted for factors shown in Table 1, and trial arm [↑](#footnote-ref-3)
